# The Impact of Intermittent Umbilical Cord Occlusions on the Inflammatory Response in Pre-Term Fetal Sheep

**DOI:** 10.1371/journal.pone.0039043

**Published:** 2012-06-20

**Authors:** Andrew P. Prout, Martin G. Frasch, Ruud Veldhuizen, Rob Hammond, Brad Matushewski, Bryan S. Richardson

**Affiliations:** 1 Department of Obstetrics and Gynaecology, The University of Western Ontario, London, Ontario, Canada; 2 Department of Physiology and Pharmacology, The University of Western Ontario, London, Ontario, Canada; 3 Department of Pathology, The University of Western Ontario, London, Ontario, Canda; Beth Israel Deaconess Medical Center, Harvard Medical School, United States of America

## Abstract

Fetal hypoxic episodes may occur antepartum with the potential to induce systemic and cerebral inflammatory responses thereby contributing to brain injury. We hypothesized that intermittent umbilical cord occlusions (UCOs) of sufficient severity but without cumulative acidosis will lead to a fetal inflammatory response. Thirty-one chronically instrumented fetal sheep at ∼0.85 of gestation underwent four consecutive days of hourly UCOs from one to three minutes duration for six hours each day. Maternal and fetal blood samples were taken for blood gases/pH and plasma interleukin (IL)-1β and IL-6 levels. Animals were euthanized at the end of experimental study with brain tissue processed for subsequent counting of microglia and mast cells. Intermittent UCOs resulted in transitory fetal hypoxemia with associated acidemia which progressively worsened the longer umbilical blood flow was occluded, but with no cumulative blood gas or pH changes over the four days of study. Fetal arterial IL-1β and IL-6 values showed no significant change regardless of the severity of the UCOs, nor was there any evident impact on the microglia and mast cell counts for any of the brain regions studied. Accordingly, intermittent UCOs of up to three minutes duration with severe, but limited fetal hypoxemia and no cumulative acidemia, do not result in either a systemic or brain inflammatory response in the pre-term ovine fetus. However, fetal IL-1B and IL-6 values were found to be well correlated with corresponding maternal values supporting the placenta as a primary source for these cytokines with related secretion into both circulations. Female fetuses were also found to have higher IL-1β levels than males, indicating that gender may impact on the fetal inflammatory response to various stimuli.

## Introduction

Study in the ovine fetus with repetitive umbilical cord occlusions (UCOs) leading to severe acidemia as might be seen clinically during human labour, has shown an inflammatory response with an increase in plasma IL-1 β levels at the time of maximal acidosis, and an increase in microglia and mast cells within the brain as measured 24 hours thereafter [1]. The stimulus for this inflammatory response likely involves placental hypoxia and/or hypoperfusion both of which lead to increases in cytokine expression/production within the placenta [2], [3] supporting the contention that reduced umbilical blood flow due to cord compression leads to an increased release of inflammatory cytokines. Additionally, rapid alterations in fetal oxygenation with UCOs leading to cerebral ischemia-reperfusion [4], [5] may disrupt oxidative balance, with an increase in reactive oxygen species as a further stimulus to local inflammation within the brain [6], [7]. This inflammatory response with repetitive UCOs might then play a contributing role to perinatal brain injury during cord-related birth asphyxia given the evidence for such with fetal (neonatal) inflammation resulting from perinatal infection [8].

Variable fetal heart rate (FHR) decelerations suggesting umbilical cord compression and resultant fetal hypoxemia are seen clinically in 2% to 10% of antenatal FHR recordings near-term [9]–[11] and have been associated with increased risk for nuchal cord at delivery and adverse neonatal outcome. In this regard, the association of infants with a symptomatic and/or tight nuchal cord at delivery and the later development of subclinical neurodevelopmental deficits [12] and cerebral palsy [13] implicate a role for chronic intermittent UCO insults antenatally in longer-term injury to the brain. Moreover, we have previously shown in the ovine fetus that intermittent UCO over a four day period does lead to a low level of necrotic appearing cells in the gray matter [14] and a marginal increase in apoptotic appearing cells in the hippocampus [15].

We have therefore used the chronically catheterized ovine fetus to test the hypothesis that intermittent UCOs of sufficient severity but without cumulative acidosis to ensure survival and thereby relevance to antenatal study, will also lead to an inflammatory response. The pro-inflammatory cytokines IL-1β and IL-6 have been determined as measures of systemic inflammation since these cytokines play a prominent regulatory role in the perinatal inflammatory response to infection and with newborn hypoxic-ischemic encephalopathy [8], [16]–[18]. The distribution of microglia and mast cells within the brain have been determined as measures of local inflammation since these cellular components also play a prominent role in the inflammatory response with fetal/neonatal infection and/or hypoxia [8], [19]–[21]. The relationship of fetal cytokine levels to maternal cytokine levels and the role of fetal gender were also assessed, since the placenta may be a common source of cytokines for both circulations and given reports whereby fetal-placental immune responses may be gender based as in later life [22]–[25].

**Figure 1:**
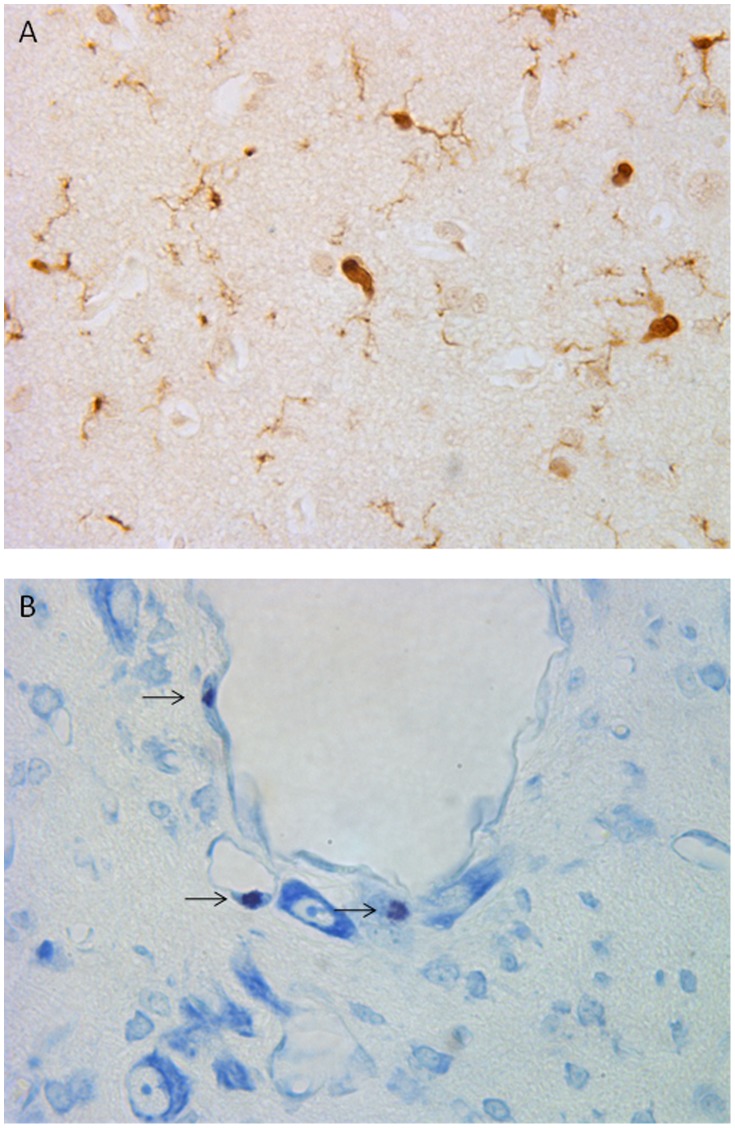
Photomicrographs taken at 60× magnification showing A, microglia in the hippocampus identified by anti-IBA1 contiguous cytoplasmic staining and B, mast cells in the thalamus identified by toluidine blue staining and characteristic morphology with the presence of large metachromatic secretory granules filling the cytoplasm and a unilobular ovoid nucleus (denoted by arrows).

## Methods

### Ethics Statement

This study was carried out in strict accordance with the recommendations for the care and use of laboratory animals by the Canadian Council on Animal Care. The protocol was approved by the Committee on the Ethics of Animal Experiments of the University of Western Ontario (Permit Number: 2005-061-09; ‘Fetal Brain Development: The Impact of Acute and Chronic Hypoxia’).

**Table 1:** Fetal plasma cytokine measurements (pg/mL).

	Day 1		Day 4	
	pre UCO 1	post UCO 6	pre UCO 1	post UCO 6
IL-1β				
Controls (8)	508±95	477±90	423±102	526±94
Mild UCO (6)	640±126	672±153	527±70	509±95
Mod UCO (5)	645±119	657±196	755±158	874±173
Severe UCO (7)	1017±264	1032±219	846±258	1135±309
IL-6				
Controls (6)	336±152	328±149	321±150	321±150
Mild UCO (5)	271±178	253±165	264±171	252±174
Mod UCO (4)	353±239	278±181	309±174	318±185
Severe UCO (7)	478±169	501±203	394±168	335±135

Data are presented as mean ± SEM. Animal numbers for each of the groups are shown in parentheses.

### Surgical Preparation

Thirty-one pre-term (113–117 days’ gestation) fetal sheep of mixed breed were surgically instrumented (term = 145 days). The anesthetic and surgical procedures and postoperative care of the animals have previously been described [26]. Briefly, using sterile technique under general anesthesia (1 g thiopental sodium in solution intravenously (IV) for induction; Abbott Laboratories Ltd., Montreal, Canada; followed by 1% to 1.5% halothane in O_2_ for maintenance), a midline incision was made in the lower abdominal wall, and the uterus was palpated to determine fetal number and position. The upper body of the fetus and proximal portion of the umbilical cord were exteriorized through an incision in the uterine wall. Polyvinyl catheters (Bolab, Lake Havasu City, AZ) were placed in the right and left brachiocephalic arteries, and the right brachiocephalic vein. An inflatable silicone occluder cuff (OCHD16; In Vivo Metric, Healdsburg, CA) was positioned around the proximal portion of the umbilical cord and secured to the abdominal skin. Once the fetus was returned to the uterus, a catheter was placed in the amniotic fluid cavity and subsequently in the maternal femoral vein. Antibiotics were administered intra-operatively to the mother, (0.2 g trimethoprim and 1.2 g sulfadoxine, Schering Canada Inc., Pointe-Claire, Canada), fetus and amniotic cavity (1 million IU penicillin G sodium, Pharmaceutical Partners of Canada, Richmond Hill, Canada). The uterus and abdominal wall incisions were sutured in layers and catheters exteriorized through the maternal flank and secured to the back of the ewe in a plastic pouch.

**Figure 2:**
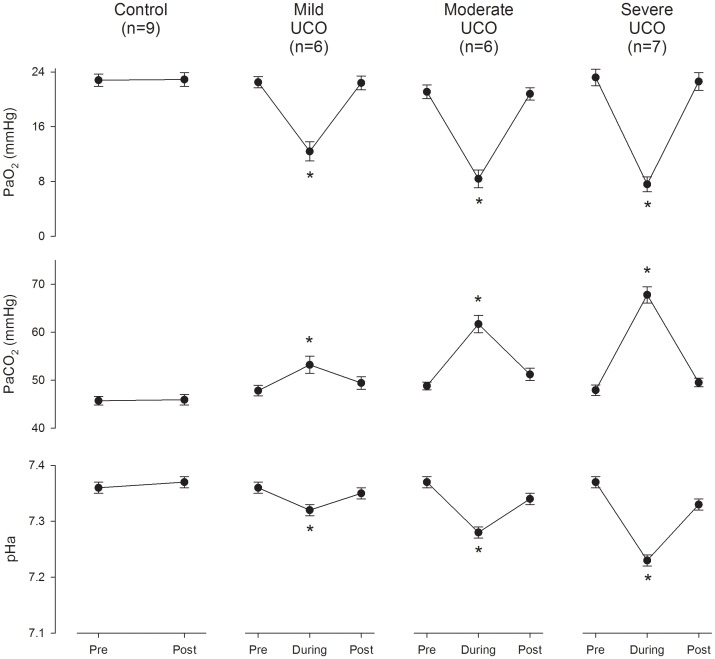
Fetal arterial blood gases and pH averaged for all of the cord occlusions as studied (see methods) for each of the control and mild, moderate, and severe UCO groups. Values are means ± SEM. Asterisk, p<0.01 compared with respective pre-occlusion values.

Animals were allowed a 3–4 day postoperative period prior to experimentation, during which the antibiotic administration was continued. Arterial blood was sampled each day for evaluation of fetal condition and catheters were flushed with heparinized saline to maintain patency. Animal care followed the guidelines of the Canadian Council on Animal Care and was approved by the University of Western Ontario Council on Animal Care.

### Experimental Procedure

Animals were studied over a four day experimental period of intermittent UCOs of varying duration. A computerized data acquisition system was used to record pressures in the fetal brachiocephalic artery and amniotic cavity, which were monitored continuously through the 4 day study. After a 2 hour baseline control period which began at ∼0800, six intermittent UCOs were performed over a six hour period, followed by a 1 hour recovery period on each of the four experimental days. UCO was induced by complete inflation of the occluder cuff with ∼5 mL saline solution which was previously determined by visual inspection and testing at the time of surgery. Animals were arbitrarily placed into either control, mild, moderate or severe UCO groups. The control group (n = 9) received no occlusions, while the mild UCO group (n = 6) received cord occlusions of 1 minute duration every hour, the moderate UCO group (n = 8) received cord occlusions of 2 minute duration every hour, and the severe UCO group (n = 8) received cord occlusions of 3 minute duration every hour. Maternal venous and fetal arterial blood samples (3 mL) were obtained five minutes before the first cord occlusion on days one and four, and five minutes after the last cord occlusion on days one and four. Fetal arterial blood samples (1 mL) were additionally obtained five minutes before, at the end of, and five minutes after the first and last cord occlusions on days one and four. Maternal and fetal 3 mL blood samples were immediately spun at 4°C (4 minutes, 4000 g-force; Beckman TJ-6, Fullerton, CA) and the plasma decanted and stored at −80°C for subsequent cytokine analysis. Fetal 1 mL blood samples were analyzed for blood gas values and pH with an ABL-725 blood gas analyzer (Radiometer, Copenhagen, Denmark) with temperature corrected to 39°C.

After the 1 hour recovery period on day four, the ewe and fetus were killed with an overdose of barbiturate (30 mg pentobarbital sodium, Fatal-Plus; Vortech Pharmaceuticals, Dearborn, MI) and a post mortem was carried out during which fetal gender and weight were determined, and the location and function of the umbilical cord occluder cuff were confirmed. The fetal brain was then perfusion-fixed with 500 mL of cold saline followed by 500 mL of 4% paraformaldehyde and processed for histochemical analysis as we have previously reported [14].

**Figure 3:**
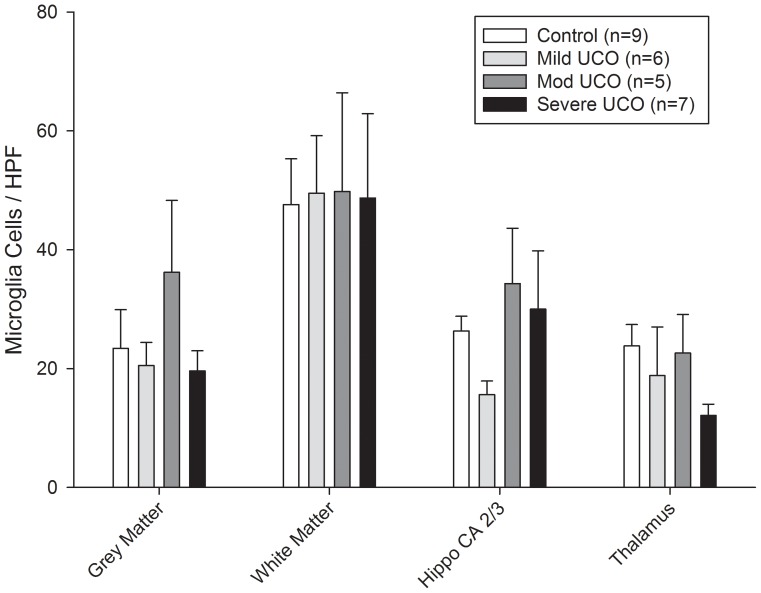
Bar graph of microglia immunoreactivity expressed as the number of identified microglia cells staining IBA positive (see methods)/high power field (HPF) in the different regions of the control (open bars) and mild, moderate, and severe UCO (hatched/shaded bars) groups of animals. Values are means ± SEM. Hippo  =  hippocampus.

### Plasma Cytokine and Tissue Histochemical Analysis

An ELISA was used to analyse in duplicate the concentrations of IL-1β and IL-6 in fetal arterial and maternal venous plasma samples as we have previously reported [1]. IL-1β and IL-6 standards were purchased from the University of Melbourne, Centre of Animal Biotechnology, Melbourne, Australia. Mouse anti-ovine IL-1β (MAB 1001) and IL-6 (MAB 1004) antibodies and rabbit anti-ovine IL-1β (AB 1838) and IL-6 (AB 1889) polyclonal antibodies were purchased from Chemicon International, Temecula, CA.

**Figure 4:**
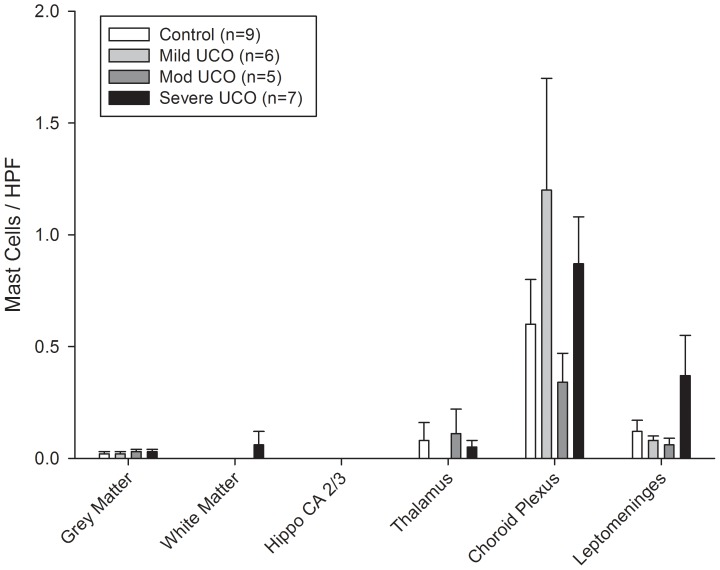
Bar graph of mast cell distribution expressed as the number of identified mast cells staining toluidine blue positive and with characteristic morphology (see methods)/high power field (HPF) in the different regions of the control (open bars) and mild, moderate, and severe UCO (hatched/shaded bars) groups of animals. Values are means ± SEM. Hippo  =  hippocampus.

The presence of microglia in brain tissue was determined by avidin-biotin-peroxidase complex enhanced immunohistochemistry (Vectastain Elite; Vector Laboratories, Burlingame, CA) as we have previously reported [1]. To reduce staining variability, all immunohistochemistry was performed on the same day with the same batch of antibody and solutions. Tissue sections were incubated with an anti-IBA1 rabbit polyclonal antibody (1∶500, Wako Industries, Richmond, VA) which has been reported to be a robust marker for microglia in human and animal studies [27], [28] with detection of bound antibody obtained following incubation in Cardassian DAB Chromogen (Biocare Medical, Concord, CA). The presence of mast cells in brain tissue was determined using histological and morphological assessment techniques after tissue sections were stained in 0.1 M HCL with toluidine blue [1].

Brain regions that were selected from each animal for analysis were taken from a coronal section of blocked cerebral hemisphere tissue at the level of the mamillary bodies and included the parasaggital and convexity cerebral gray matter and leptomeninges, periventricular white matter, thalamus, choroid plexus and the combined CA2 and CA3 regions of the hippocampus. Each of the gray matter regions was further divided into sub-regions combining layers 1, 2, and 3 and layers 4, 5, and 6. After showing no significant difference between these subregions, all layers were combined to represent the gray matter. Image analysis was performed with a transmitted light microscope (Leica DMRB, Leica-Microsystems, Wetzler, Germany) at 40× magnification. Positive microglia cell immunostaining was quantified with an image analysis program (Image Pro Plus 6.0, Media Cybernetics, Silver Spring, MD). The image analysis system was first calibrated for the magnification settings that were used, and thresholds were established to provide even lighting and no background signal. Six high-power field (HPF) photomicrographs (HPF area = 7 cm^2^) per brain region/subregion per animal were collected as a 24 bit RGB colour modeled image. The same illumination setting was applied to all images for all of the brain regions, therefore allowing for comparison within each brain region (i.e. control versus intermittent cord occlusion animal groups), and between brain regions (i.e. gray matter versus white matter). For the microglia analysis, and using the Image Pro Plus’ RGB colour range selection tool, colour sampling of positive DAB stained areas were obtained from multiple brain regions of control and UCO animals, and tested for specificity against the negative control. Appropriate ranges of colour were selected showing positive contiguous cytoplasmic staining as a criteria for microglia cell count scoring which were then applied uniformly to calibrated images for all brain regions (Figure 1). Scoring was performed in a blinded fashion to experimental groups. For mast cell analysis, scoring was performed manually based upon positive stain and characteristic morphology with the presence of large metachromatic secretory granules filling the cytoplasm which depend on the presence of sulfonated proteoglycans such as heparin, and a unilobular ovoid nucleus (Figure 1). Scoring was again performed in a blinded fashion to experimental groups.

### Data Analysis

A one-way repeated measures ANOVA with a SNK correction for multiple comparisons were used to compare maternal and/or fetal blood gas, pH and cytokine values for each of the animal groups at the different time points over the 4 day study. A two-way repeated measures ANOVA was used to test for differences in microglia and mast cell counts between brain regions, and the different animal groups. Correlation analyses were performed using Spearman correlation coefficient (SigmaStat, Systat Software, Inc., San Jose, CA). All values are expressed as means ± SEM. Statistical significance was assumed for p<0.05. Not all measurements were obtained for each animal studied (see Table 1, and Figures 2, 3 and 4).

## Results

All animals studied had normal PaO_2_, PaCO_2_ and pH_a_ values when measured five minutes before the first UCO on day one which averaged 22.8±0.4 mmHg; 48.4±0.4 mmHg; and 7.37±0.01, respectively. Two of the moderate UCO animals and one of the severe UCO animals showed a worsening acidemia on day four of study and were therefore analyzed separately. The remaining animals showed no overall change in their 5 minute pre UCO blood gas and pH values, nor in the blood gas/pH response with each UCO as measured on days one and four of study. Accordingly, these were averaged for the respective groups as shown in Figure 2. As such, intermittent UCOs as studied for the mild, moderate, and severe UCO groups resulted in a transitory decrease in fetal PaO_2_ by ∼10, 13, and 16 mmHg; in fetal pH_a_ by ∼0.04, 0.09, and 0.14; and a transitory increase in fetal PaCO_2_ by ∼5, 13, and 20 mmHg, respectively (all p<0.01), but with a rapid return toward pre-occlusion values as measured 5 minutes after release of the occluder cuff (Figure 2). The three animals with worsening acidemia on day four of study with pH_a_ values at 7.23, 7.23, and 7.28 as measured 5 minutes after the last UCO, were additionally moderately hypoxic this day with PaO_2_ values at 12.0, 16.3, and 16.9 mmHg, respectively, prior to the onset of UCOs.

### Plasma Cytokine Measurements

Plasma cytokine measurements at the selected time points from fetal arterial blood sampling are shown in Table 1. Fetal IL-1β values showed considerable variance across the animal groups, being lowest in the control animals and highest in the severe UCO animals. However, for each of the animal groups there was no significant change in these values over the four days of study and thereby in relation to intermittent UCO insults. Fetal IL-6 values were also variable across the animal groups and again showed no significant change over the four days of study and thereby in relation to the UCO insults. Maternal IL-1β and IL-6 values also showed no significant change from that prior to the first UCO on day 1, and overall averaged 450±66 and 365±85 pg/mL, respectively, for all of the animal groups. However, maternal IL-1β and IL-6 values were again highest in the severe UCO animals at 522±97 and 572±176 pg/mL, respectively.

Baseline cytokine findings for individual animals as measured prior to the first UCO on day 1 were analyzed for significant correlations to further assess the relationship of fetal to maternal values, and of IL-1β to IL-6 values. Fetal IL-1β and IL-6 values at baseline were found to be well correlated with corresponding maternal IL-1β and IL-6 values, r = 0.75 and 0.65, respectively (both p<0.001). Likewise, fetal and maternal IL-1β values at baseline showed a modest correlation with corresponding IL-6 values, r = 0.44 (p<0.05) and 0.62 (p<0.01), respectively. Moreover, there was a gender effect with female fetuses showing higher IL-1β levels than males, 858±117 versus 481±101 pg/mL (p<0.05) as did their mothers, 453±83 versus 324±89 pg/mL, although this was not statistically significant. This accounted in part for the higher fetal IL-1β values in the severe UCO animals which had a disproportionate number of female fetuses at 5 out of 7 in comparison to the other animal groupings, at 50% or less.

The two animals with fetal pH_a_ falling to 7.23 on the last day of study both showed an apparent systemic inflammatory response when compared to baseline control values with one showing an ∼3 fold increase in IL-1β levels to 2618 pg/mL, while the other showed an ∼8 fold increase in IL-6 levels to 1026 pg/mL.

### Histochemical Scoring for Microglia and Mast Cells

Microglia immunoreactivity was analyzed at the end of the four days of intermittent UCO as a measure of local inflammatory response within the brain. In the control group animals regional differences were evident with the microglia cell counts in the white matter at 47.6±7.7 cells/HPF higher than that in all other regions (p<0.05) (Figure 3). For the UCO groups of animals the microglia cell counts were likewise highest in the white matter, but there were no significant differences from respective control values for any of the brain regions studied (Figure 3).

Mast cell distribution was analyzed at the end of the four days of study as a second measure of the local inflammatory response within the brain. In the control group animals regional differences were again evident with the mast cell counts in the choroid plexus while extremely low at 0.6±0.2 cells/HPF, still higher than that in all other regions (p<0.05) (Figure 4). For the UCO groups of animals the mast cell counts were likewise highest in the choroid plexus, but there were no significant differences from respective control values for any of the brain regions studied (Figure 4).

None of the three UCO animals with worsening acidemia on day four of study showed notable microglia cell changes, although the two animals with fetal pHa falling to 7.23 did show high mast cell counts in the choroid plexus at 2.7 and 1.9 cells/HPF.

## Discussion

Intermittent UCOs as studied resulted in transitory fetal hypoxemia with associated acidemia which was most pronounced in the severe UCO group as expected with umbilical blood flow occluded the longest. The UCO induced acidemia can be attributed to the fetal hypercapnia also noted, as well as related increases in lactate levels likely indicating the onset of anaerobic metabolism by some fetal tissues including carcass and muscle with the redistribution of blood flow away from these tissues as we have previously shown [4]. However, while UCO insults resulted in moderate to severe degrees of fetal hypoxia and hypercapnia, for the most part there was complete recovery post occlusion with no cumulative blood gas or pH changes observed throughout the 4 days of study either at baseline pre-occlusion or during the UCOs. This would indicate that intermittent UCO up to 3 minutes in duration are unlikely to have a residual impact on umbilical blood flow or cotyledonary blood gas exchange. Exceptions were the two moderate and one severe UCO group animals with moderate hypoxemia and worsening acidemia on day four of study and indicating some degree of altered uteroplacental gas exchange, whether related to the intermittent UCOs or otherwise.

Fetal arterial IL-β and IL-6 values showed no significant change as measured pre-term over the four days of intermittent UCO insults regardless of their severity. This is in contrast to studies in humans where birth asphyxia with concerning hypoxic-acidemia has been shown to result in elevated cytokines in umbilical cord blood including IL-6 [17], [18], and to our study in near term fetal sheep where repetitive UCO leading to severe acidemia resulted in a 2 fold increase in plasma IL-1β values [1]. As such, intermittent UCO in the absence of worsening acidemia is not a sufficient stimulus to evoke a systemic inflammatory response with increases in IL-1β and IL-6 plasma values. This is consistent with the short half-life of cytokines and need for repeated stimuli for continued production [8]. It is thus of interest that the two animals with sustained acidemia and pH_a_ falling to 7.23 on the last day of study both showed a systemic inflammatory response with increases in IL-1β and IL-6 plasma levels, and indicating that sustained hypoxia with worsening acidemia are sufficient stimulus for such response.

Fetal IL-1β and IL-6 values at baseline for individual animals were well correlated with corresponding maternal values. This is to be expected if the placenta is a primary source for these cytokines and with related secretion into both circulations, since IL-1β and IL-6 are unlikely to cross the cotyledonary placenta given their molecular weight [29]. Accordingly, the variance in IL-1β and IL-6 plasma values across the animals may relate in part to the initial surgical preparation and recovery from such, and the extent to which utero-cotyledonary tissues were traumatized with resultant inflammation leading to differing secretion rates for these cytokines. Fetal and maternal IL-1β values at baseline also showed a modest correlation with corresponding IL-6 values, which is again to be expected since IL-1β is known to stimulate expression of IL-6 in various cell types including leukocytes and endothelium through autocrine, paracrine and/or endocrine mechanisms [8], [30]. Moreover, this observed relationship might also reflect the joint involvement of these two cytokines in the utero-cotyledonary inflammatory response to surgery and related secretion into both circulations. Of additional interest, female fetuses were found to have higher IL-1β levels than males, indicating that gender may impact on the fetal inflammatory response to various stimuli, in this case presumably the surgical manipulation several days earlier. This is consistent with the finding in patients delivering preterm whereby those with female infants are more likely to show placental inflammation [31] and have higher amniotic fluid IL-1 receptor antagonist levels as a homeostatic counter measure [22], but to our knowledge there has been little other study of fetal gender and immune responses. Nonetheless, there has been considerable human and animal-based study in adults of gender effects on immune responses. For the most part these support the contention of enhanced immune responses in females compared to males which is likely hormonally mediated [23], [24], although this may well depend upon the immune response trigger and be different for infectious versus aseptic inflammatory stimuli [23]–[25].

Microglia and mast cell counts were found to be highest in the white matter and choroid plexus, respectively, as we have previously reported for the ovine fetus near term [1], but there was no evident impact of the intermittent UCO insults on these cell counts for any of the brain regions studied. This again is in contrast to our study in near term fetal sheep with repetitive UCO leading to severe acidemia which resulted in a ∼2 fold increase in microglia cell counts in the white matter and hippocampus, and a ∼2 fold increase in mast cell counts in the choroid plexus and de novo appearance in the thalamus [1]. As such, intermittent UCOs as studied and known to result in cerebral ischemia-reperfusion [4], [5], but in the absence of worsening acidemia and evident systemic inflammation, are not a sufficient stimulus to evoke a local inflammatory response within the brain with increases in microglia and mast cell counts. This would support our contention that an increase in circulating cytokines is required to modulate the brain inflammatory response given their ability to increase blood-brain barrier permeability to macrophages and other cellular and molecular inflammatory mediators [8], [19]. In this regard, the two animals with worsening acidemia on the last day of study and apparent systemic inflammation also showed high mast cell counts in the choroid plexus as further support for the role of circulating cytokines in the brain inflammatory response.

We have previously shown that intermittent UCOs of 90 second duration in both preterm and near term fetal sheep can lead to a low level of necrotic appearing cells in the gray matter [14] and a marginal increase in apoptotic appearing cells in the hippocampus [15]. This could involve inflammatory mediators which can become injurious by activating apoptotic and necrotic pathways in the developing brain [8], [32] and might be increased systemically in response to placental hypoxia and/or hypoperfusion with cord compression [2], [3], and locally within the brain in response to ischemia-reperfusion with an increase in reactive oxygen species [4]–[7]. We have now determined that intermittent UCOs of up to 3 minutes duration with severe, but limited fetal hypoxemia and no cumulative blood gas or pH changes as studied over a 4 day period, do not result in either a systemic or local inflammatory response within the brain. To the extent that intermittent cord compression does occur over the latter part of human pregnancy [9]–[11] and relates to adverse neurodevelopment [12], [13], the present findings would indicate that this is unlikely to involve inflammatory pathway mechanisms. We have also determined that the cotyledonary placenta in sheep is likely to be a primary source of inflammatory cytokines with related secretion into both the maternal and fetal circulations. Moreover, this linkage between maternal and fetal immune responses appears to be impacted by fetal gender, being heightened in females and warrants further study.
